# Odontolithus

**Published:** 1875-04

**Authors:** J. M. Lucas

**Affiliations:** Middleport, Ohio


### ODONTOLITHUS.

BY J. M. LUCAS, M. D., MIDDLEPORT, OHIO.

Odontolithus (from *odonto* and *λιθος*, a stone) includes all of those calcareous deposits on the teeth, which are commonly designated tartar, ranging in color from a white to a dark brown and even to a black ; in texture, from a very soft, that may be removed by the tooth-brush, to that which is nearly or quite as hard as the tooth itself—which can be removed with an instrument only with much difficulty.

The color of the deposit shows its density, and the time employed in formation—the lighter colored being porous and of rapid formation, while the darker varieties are formed more slowly and have a closer texture.

According to Berzelius, tartar is composed of—

| | |
|---|---|
| Earthy Phosphates, | 79.0 |
| Salivary Mucus, | 12.5 |
| Ptyalin, | 1.0 |
| Animal Matter, | 7.5 |
| | 100 |

This analysis can only be taken as an approximate one, for no two specimens give precisely the same analytical reaction. While that deposit found on the lower incisors will give a large per centage of phosphate of lime and organic matter, that found near the orifice of Steno's duct will contain a large amount of carbonate of lime and but little of the phosphate, or of the organic matter.

The oral secretions give, normally, an alkaline reaction ; but the mucus which is secreted by the gingival mucous membrane, containing a large amount of albuminoid matter, owing to its tenacity, becomes lodged around and upon the teeth, where it undergoes fermentation, becoming acrid precipitates the salts from the saliva, forming a lodgment for both animal and vegetable parasites, chief among which is the phyto-parasite *Septothrix buccalis*, which, with the epithelial cells, forms a kind of cement which unites the mass more closely together and gives to it its color.

Tartar is most frequently found upon those teeth which come most directly under the influence of the saliva, viz: the outer surface of the upper second bicuspids and first molars, the inner and outer surfaces of the lower incisors and cuspids, upon those teeth which have no antagonists and those which, being tender, are little used or cleaned. With those persons who have acquired the habit of chewing on one side exclusively, the teeth which are not used will become covered, while those of the opposite side, which are made to perform the office of mastication, will be comparatively free.

The deposit of tartar on the teeth appears in the shape of a wedge, with its base directed toward the gums, presenting a sharp, uneven surface, which keeps up a constant irritation, while the sides are kept smooth by friction with the tongue and the buccal and labial mucous membrane.

The deposit produces no injurious effects upon the teeth directly, but its encroachments on their fangs cause an absorption of their alveoli and a recession of their gums, depriving the teeth of their support, in consequence of which they loosen, become painful, and are eventually lost. Another evil result is, suppuration, with exfoliation of the gums. Large deposits of tartar upon the teeth are, oftentimes, the cause of very offensive breath.

Some think that tartar is a preventive of caries. This, no doubt, is a mistaken idea. The saliva, in mouths that are subject to large deposits, is strongly alkaline, and it is to this, I think, rather than to the tartar, that their exemption from caries is due.

There is a dark deposit, found on the teeth of habitual smokers, of a sooty nature, which is generally classed under this head ; also one of a greenish color, met with on the teeth of young persons, which is of an erosive character, somewhat more allied to caries, and is probably started by the putrefaction of the enamel cuticle, (Nesmith's membrane), and has nothing in common with tartar.

A judicious use of the brush will keep all of these deposits from the teeth ; but if they have been allowed to accumulate, they should be removed with instruments. This operation is, in most instances, of more or less difficulty, and requires a nicety of manipulation which can only be acquired by time and practice. After this, they should be polished with a wooden polisher and pulverized pumice-stone, or something of that nature, for if the teeth be left with particles of tartar remaining, they form the nuclei for the formation of new deposits. Any acid that will dissolve it, will also injure the teeth. It has been found that the tincture of iodine softens it somewhat, and causes it to loosen its hold upon the tooth. I have used it, in connectien with instruments, with good results.

It is essential, in our manipulations, to avoid wounding the enamel and the gums; the latter, however, is very difficult, owing to the extreme sensitiveness and congested condition which always exists with large deposits.

Many other points regarding odontolithus might be dwelt upon in a paper prepared for dentists exclusively, yet which would hardly be appropriate for a body of medical gentlemen.

